# Physicochemical Properties, Antioxidant Activity, and High-Performance Thin-Layer Chromatography Profiling of Propolis Samples from Western Australia

**DOI:** 10.3390/plants13141919

**Published:** 2024-07-11

**Authors:** Juliane Achenbach, Nicola Deyerling, Mariana Mello dos Santos, Sharmin Sultana, Md Khairul Islam, Cornelia Locher

**Affiliations:** Division of Pharmacy, School of Allied Health, The University of Western Australia, Curnow Building M315, Crawley, WA 6009, Australia; juliane.achenbach@uwa.edu.au (J.A.); nicola.deyerling@uwa.edu.au (N.D.); mariana.mellodossantos@research.uwa.edu.au (M.M.d.S.); sharmin.sultana@research.uwa.edu.au (S.S.); khairul.islam@uwa.edu.au (M.K.I.)

**Keywords:** propolis, HPTLC, *Apis mellifera*, antioxidant activity, phenolic determination, physiochemical properties, Western Australia

## Abstract

This study reports on the physicochemical and antioxidant properties of propolis samples from various regions across Western Australia and identifies some phenolic constituents using high-performance thin-layer chromatography (HPTLC). Total phenolic content (TPC) was determined using a modified Folin–Ciocalteu assay, and antioxidant activity was investigated with the Ferric Reducing Antioxidant Power (FRAP) assay and also visualised and semi-quantified by HPTLC-DPPH analysis. TPC values ranged from 9.26 to 59.3 mg gallic acid equivalent/g of raw propolis and FRAP assay data from 4.34 to 53.8 mmol Fe^2+^ mmol/kg of raw propolis, although some of these variations might be related to differences in extraction yields obtained with 70% ethanol. The presence of luteolin, taxifolin, naringenin, and 4-hydroxyphenylacetic acid was confirmed based on a comprehensive, validated matching approach against an HPTLC-derived database. The findings of the study highlight the importance of future research on the chemical composition and bioactivity of Western Australian propolis.

## 1. Introduction

Propolis is a resinous hive product made by honeybees from beeswax and saliva mixed with exudates of plant tissues such as leaf or flower buds, wounds in the bark or stems, and also leaf glands [1,2]. As these plant exudates are produced in response to microbial infection or insect attack, they tend to contain substances that can act as chemical defence against bacteria, fungi, or viruses [2,3]. Honeybees take advantage of these bioactive phytochemicals and therefore use propolis as a construction material for blocking cracks in their hives in order to reduce the chances of microbial hive contamination and to deter intruders [3,4].

The interest in propolis as a complementary medicine has grown in recent years due to its reported antiproliferative, antibacterial, antifungal, anti-inflammatory, and antioxidant properties [1,2,5,6]. The natural product is commercially available in various formulations which are primarily aimed at the treatment of skin and respiratory tract infections due to its antimicrobial and anti-inflammatory properties. However, its use in medical care spans wider across a range of conditions, including tumours and parasitic infections [2], although more research should underpin these clinical applications, specifically with respect to bioactive constituents and their in vitro and in vivo effects.

Propolis typically consists of substances such as resin, wax, essential oils, pollen and ‘other’ substances, which include cinnamic and phenolic acid derivatives, substituted benzoic acids, amino acids, and flavonoids [3,7]. More than 300 compounds have, to date, been reported from propolis samples collected around the world and it has been demonstrated that their pharmacological activity is predominantly influenced by phenolic constituents [2,8]. As a natural product, the specific chemical composition of each propolis sample varies and is strongly influenced by the plant exudates collected by bees [7]. In Europe, for example, bees tend to gather resin from the leaf buds of poplar (*Populus nigra* L.) and beech (*Fagus sylvatica* L.); however, when these are unavailable, they may also forage on other resin-producing plants [7,9]. Brazilian green propolis, on the other hand, has been found to be mainly produced from *Baccharis dracunculifolia* as its major plant source [10].

With its rich and often unique flora and fauna, Australia is one of the world’s megadiverse countries [5]. As the chemical composition, and with this, also the potential medicinal properties of propolis, largely rely on its botanical and geographical origins, it can be assumed that Australian propolis features a unique chemical composition that could also be used as complementary medicine [5]. However, to date, only a few studies have been carried out to identify bioactive constituents in Australian propolis using nuclear magnetic resonance (NMR) [11], thin-layer chromatography (TLC) [12], gas chromatography coupled with mass spectrometry (GC-MS) [13], and high-performance liquid chromatography (HPLC) using UV or diode array detection (DAD) [14]. This study focuses specifically on a qualitative analysis of propolis samples collected in Western Australia, including their antioxidant activity and physicochemical characteristics. It also attempts to identify some constituents in these samples. To date, no study has focused on propolis from this geographical region although Western Australia constitutes the largest state in Australia, making up about half of its land mass, and is also home to 8 of Australia’s 15 biodiversity hotspots [15].

Another novel aspect of this study is its use of high-performance thin-layer chromatography (HPTLC), which has already been demonstrated to be a useful tool in the authentication and quality control of honey [16,17,18,19,20]. HPTLC-derived fingerprints of Western Australian propolis offer researchers a novel approach to understanding its unique chemical composition, and with selective post-chromatographic derivatisation, it might even assist in the detection of antioxidant constituents that contribute to its potential health benefits [21]. HPTLC analysis is also simple, convenient, and fast as it allows to visualise and thus also compare various constituents of several propolis samples in a single run [22]. Furthermore, the data generated by HPTLC analysis of propolis samples can be matched against an established phenolic database, which has previously been successfully employed to identify a range of honey constituents [22].

In short, the aim of this study is to collate data on the physicochemical properties, total phenolic content, total antioxidant activity, and also the HPTLC profile of Western Australian propolis samples. An HPTLC-derived database is used for the identification of some propolis constituents and HPTLC-DPPH bioautography for the visualisation and semi-quantitative assessment of their antioxidant activity.

## 2. Results

After carrying out the Ferric Reducing Antioxidant Power (FRAP) assay, the total phenolic content (TPC) assay, and preliminary high-performance thin-layer chromatography (HPTLC) fingerprinting on all 32 propolis samples received, six samples were selected for an in-depth analysis based on their performance in these assays and their HPTLC fingerprint characteristics. Samples with either low TPC results or similar HPTLC fingerprints were excluded from further analysis, and the preliminary data collated on these can be found in the supplementary file.

### 2.1. Sensorial Analysis 

The results of the sensorial analysis are presented in Table 1 for the six propolis samples selected for an in-depth analysis. All six samples featured a resinous aroma and a malleable consistency, but there were some significant differences in their respective colours. P29, P14, and P18 were mainly brown, and P28 and P32 had a predominantly red colour, while P10 was brown-white.

### 2.2. pH, Oxidation Index, and Extraction Yields

The average pH was acidic with a pH value of 4.43 ± 0.30 and with pH values ranging from 4.10 to 4.90 (Table 2). These pH values were similar to those found in other studies [23,24].

The samples’ oxidation index (Table 2) was compared with the recommendations of the Brazilian Ministry of Agriculture [25]. The mean value of all samples was 14.0 s, which is less than the maximum oxidation index of 22.0 s recommended by the Brazilian Ministry of Agriculture [25]. P32 showed a significantly higher value of 30.9 s compared to the other samples and thus did not meet the criteria above. With 4.20 s, P29 showed the lowest oxidation index.

The yield of propolis in each ethanolic solution was calculated (Table 2), based on the initial amount of raw propolis used for the extraction and the undissolved material that was recovered. Highly variable results were found, ranging between 0.10% (P32) and 41.0% (P29) extraction yield.

The oxidation index and pH values were analysed using a one-way ANOVA (Table 2).

### 2.3. Total Phenolic Content (TPC) 

Table 3 shows the mean total phenolic content (TPC) of the six propolis samples selected for an in-depth analysis, expressed as mg GAE/g of raw propolis. The mean TPC values of their ethanolic extracts ranged between 9.26 ± 0.20 mg GAE/g (P32) and 59.3 ± 0.93 mg GAE/g (P29), with an overall average of 36.0 ± 19.5 mg GAE/g. The minimum recorded individual TPC was found to be 9.02 (P32) and the highest was measured in P29 as 60.0 mg GAE/g raw propolis, resulting in a range of 51.0 mg GAE/g raw propolis. The average TPC of the six ethanolic propolis extracts differed significantly when analysed using a one-way ANOVA (*p* < 0.05).

### 2.4. Ferric Reducing Antioxidant Power (FRAP) Assay 

Table 3 shows the average FRAP antioxidant activity of the six ethanolic propolis extracts selected for an in-depth analysis, expressed as mmol Fe^2+^ equivalent/kg of raw propolis. The FRAP values of the ethanolic propolis extracts ranged between 8.68 ± 0.11 (P32) and 53.8 ± 4.43 (P29) with a mean FRAP activity of 23.5 mmol Fe^2+^/kg. The minimum recorded individual FRAP result was 8.57 (P32), and the maximum of 58.1 mmol Fe^2+^/kg was measured in P29, resulting in a range of 49.5 mmol Fe^2+^/kg raw propolis. A one-way ANOVA analysis demonstrated a non-significant difference between P10 and P28 (*p* = 0.40) and P14 and P18 (*p* = 0.07). However, there was a significant difference (*p* < 0.05) between the means of all other ethanolic propolis extracts. Additionally, a high correlation of 0.83 was observed between FRAP antioxidant activity and TPC. Similar correlations between FRAP and TPC values have previously been documented for a range of honey products [16] including propolis [26,27], indicating that the antioxidant activity of the propolis samples is strongly related to their phenolic compounds.

### 2.5. HPTLC Fingerprinting 

**Figure 1:**
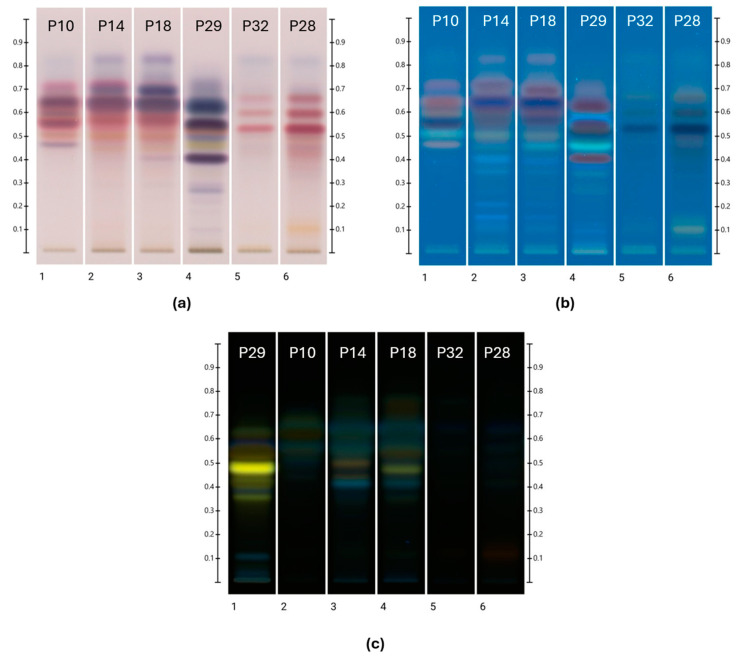
HPTLC profile of ethanolic propolis extracts. Plate images obtained under (**a**) transmittance in white light after derivatisation with VSA; (**b**) 366 nm after derivatisation with VSA; and (**c**) 366 nm after derivatisation with NP-PEG.

The HPTLC images obtained for the six selected ethanolic propolis extracts after derivatisation with either vanillin sulfuric acid (VSA) or natural product/polyethylene glycol (NP-PEG) spraying reagent are shown in Figure 1. Reflecting the respective local flora, propolis samples from similar regional origins also showed similarities in their HPTLC profile.

Except for an unresolved band at R_f_ 0.00, the obtained R_f_ values ranged from 0.100 to 0.765. The colour of each band (RGB value) was converted into corresponding hue values following analysis at 254 and 366 nm after development, as well as at 254 nm and at white light after derivatisation with VSA spraying reagent and also at 254 nm and 366 nm after derivatisation with NP-PEG. The hue values obtained at 254 nm after development ranged from 135° to 142°, representing green hues, and those obtained at 366 nm after development ranged from 196° to 212° varying from cyan blue to blue hues. After derivatisation with NP-PEG, hue values at 366 nm ranged between 60.6° and 194°, which reflects a broad range of colour hues (e.g., yellow, green, turquoise, and cyan blue), whereas derivatisation with VSA spraying reagent and analysis at 366 nm produced hue values between 198° and 238°, thus mostly blue or cyan blue colour hues. The plates were also analysed with white light in transmittance mode after the derivatisation with VSA, which yielded hue values from 1.39° to 351°, which translates into red, orange, and scarlet colours.

UV Vis spectra were obtained by scanning the plate from 190 to 900 nm. Most of the λ_max_ of the samples were detected around 254 nm and between 361 and 380 nm. The fluorescence spectra were obtained by scanning the plate from 190 to 380 nm, and most samples were found to have λ_max_ values ranging between 208 and 260 nm.

### 2.6. Phenolic Constituent Identification in Propolis

A database established by Lawag et al. [22] was used for the identification of the unknown bands. For this, R_f_ values and colours, as well as fluorescence and UV Vis spectra of bands of interest (absorbance (AU) > 0.05), were recorded and compared with the data of the database’s standards. In brief, the search criteria were applied as follows [22]: The initial identification of potential matches was based on an R_f_ value (±0.05), followed by a comparison of colour hues (±60°) after derivatisation with VSA reagent and also NP- PEG. This was followed by a comparison of λ_max_ values (±15 nm) in the respective fluorescence spectra (190–380 nm), followed by a comparison of λ_min_ and λ_max_ values in the respective UV-Vis spectra after development (190–900 nm) and after derivatisation (250–500 nm after derivatisation with NP-PEG or VSA). The threshold for this step in the database filtering was set at ±15 nm before and ±60 nm after derivatisation. To confirm a match with the resulting reduced list of potential database hits, a spectral matching approach was adopted [22]. The different HPTLC fingerprints used for the identification of phenolic constituents are shown in Figure 1.

After the analysis of over 60 bands of interest in the investigated propolis extracts, it was not possible to find a match for every band; however, some bands could be identified. Their key HPTLC characteristics alongside those of their matched standards are shown in Table 4 complemented by their respective spectral overlays (Table S2) which further confirm these identifications.

The identified constituents have also been found in propolis samples collected from various regions worldwide. For instance, luteolin, a flavonoid, has been documented in propolis from Mexico, Brazil, Poland, Portugal, and China. Similarly, naringenin was found in propolis from Brazil and Poland [28,29,30], whereas various derivates of taxifolin were identified in Libyan propolis [31], and compounds related to 4-hydroxyphenylacetic acid were found in Moroccan propolis [32].

### 2.7. HPTLC-DPPH

**Figure 2:**
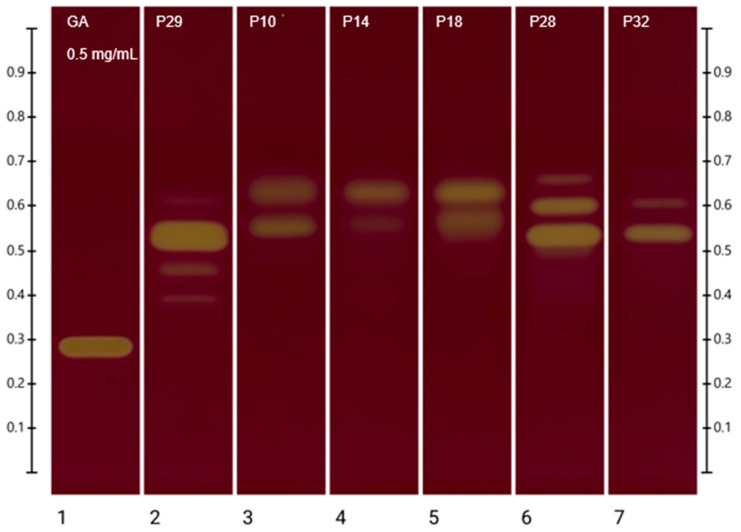
HPTLC-DPPH plate image of ethanolic propolis extracts after derivatisation with DPPH reagent.

The HPTLC-DPPH assay allows for the visualisation (Figure 2) of components that contribute to the total antioxidant activity of propolis. White light photo-documentation and scanning at 517 nm were carried out 2 h after derivatisation with the DPPH reagent to allow sufficient time for antioxidant constituents to react. The RGB value of each band was generated automatically by the HPTLC software after spraying with DPPH. The RGB value was converted to an individual hue value [22] and then used to express the level of scavenging activity in relation to that of gallic acid, which is the standard reference compound to express the total antioxidant activity in the DPPH assay (Table 5). Thus, in addition to the visualisation of strong antioxidant bands by their yellow colour (Figure 2), the antioxidant activity of bands was expressed semi-quantitatively based on the resulting colour hue [22] (Table 5).

P10 was found to have two intense bands with DPPH antioxidant activity, of which the band at R_f_ 0.554, representing naringenin (Section 2.6), had the highest impact on the antioxidant activity of the sample. Another band of high activity was recorded at R_f_ 0.630, and a band of low activity was found at R_f_ 0.479.

Two intense bands were also observed for P14 where the highest antioxidant activity was found at R_f_ 0.638. Another band of medium antioxidant activity was observed at R_f_ 0.552, which represents again the previously identified naringenin (Section 2.6).

P18 also showed two prominent bands, which both appeared to have high antioxidant activity. The highest antioxidant activity for P18 was observed at R_f_ 0.633, followed by another band of high antioxidant activity at R_f_ 0.565, which corresponds to naringenin (Section 2.6).

For P29, five bands of antioxidant activity were observed. The highest antioxidant activity was present at R_f_ 0.532, followed by other bands with medium antioxidant activity at R_f_ 0.454 and R_f_ 0.400. Other bands with low antioxidant activity were found at R_f_ 0.615 and R_f_ 0.370. Based on the previous HPTLC identification (Section 2.6), the bands at R_f_ 0.400 and R_f_ 0.454 represent luteolin and the band at R_f_ 0.370, taxifolin.

P32 showed three distinct bands with antioxidant activity. The highest activity was observed at R_f_ 0.542, followed by medium antioxidant activity at R_f_ 0.610 and low antioxidant activity at R_f_ 0.673. Unfortunately, none of these bands could be chemically identified yet.

P28 showed four bands of antioxidant activity. Those at R_f_ 0.537 and R_f_ 0.605 appear to have high antioxidant activity and represent the two constituents with the main impact on P28’s antioxidant strength, followed by a band at R_f_ 0.663 with medium antioxidant activity and one at R_f_ 0.454 with low antioxidant activity, which has been identified (Section 2.6) as 4-hydroxyphenylacetic acid (4-HPAA).

## 3. Discussion

Under most circumstances, raw propolis is not suitable for direct use in food technology or in pharmaceutical or cosmetic applications [6,33]. As it is barely soluble in water, it needs to be extracted with a suitable solvent [34]. After the evaluation of different extraction methods reported in previous studies, an ultrasonic ethanolic extraction appeared to be the most promising method to achieve a high extraction yield [6,34,35]. However, the results obtained in this study from this approach demonstrate that extraction yields can vary widely, leading to the conclusion that the respective extraction methods might need to be optimised depending on the individual propolis sample.

A comparison of TPC values obtained in this study illustrates this point as data obtained in the TPC assay ranged from 9.26 (P32) to 59.3 (P29) mg GAE/g raw propolis, thus revealing significant differences among the samples. However, this wide range of TPC levels may also be, in part, attributed to the different extraction efficiencies as P32 had by far the least concentrated extract, thus resulting in a low TPC value when expressed per g of raw propolis. Nonetheless, other studies reported TPC values for propolis from Azerbaijan ranging from 10.94 to 79.86 mg GAE/g of raw propolis [26], from Malaysia with 28.09 mg GAE/g [36], from Spain ranging from 200 to 340 mg GAE/g, and Australia ranging from 1.30 to 180.5 mg GAE/g [11]. The TPC values obtained in this study also fall within these broad ranges.

Reflecting the high correlation noted in the study between TPC and antioxidant activity, FRAP activity of P29 with 53.8 mmol Fe^2+^/kg of raw propolis was observed to be the highest amongst the investigated WA propolis samples. In turn, the lowest FRAP values were recorded for P32 with 8.68 mmol Fe^2+^/kg. Other studies reported FRAP values for propolis from Russia, Ukraine, Kazakhstan, Slovakia, and Poland to range between 60.0 ± 10.0 and 1170 ± 60.0 mmol Fe^2+^/kg [37], from Brazil with 1473 ± 72.4 mmol Fe^2+^/kg [27], and from Croatia with 100.0 to 800.0 mmol Fe^2+^/kg [38]. Compared to these studies, the FRAP results of the investigated ethanolic propolis extracts from Western Australia seem very low, although this might be related to individual experimental conditions.

The measured oxidation index reflects a trend similar to that seen in the FRAP analysis insofar as P29 had the fastest oxidation time and P32 the slowest. However, as mentioned earlier, these differences might be related to different concentrations as the extraction yield for P32 was only about 0.10% compared to the yield of P29 at 41.0%. In addition, as previous studies about propolis suggest, the botanical characteristics of the region of collection influence the characteristics of each propolis sample [1,28,39], and thus other factors such as collection time of the propolis and its floral sources as well as storage and handling conditions might also contribute to the wide variation in antioxidant activity seen in Western Australian propolis samples.

The HPTLC-DPPH assay which was carried out in this study allows for a visualisation and semi-quantitative analysis of the extracted antioxidant constituents and therefore allows for a comparison independent of the respective extraction efficiency. All analysed samples had at least two bands with medium and high antioxidant activity. While only six of these could so far be identified, it is apparent that there are many constituents in each propolis extract that contribute to its antioxidant activity. The band at R_f_ 0.63, for example, contributes a significant antioxidant activity to the propolis samples P10, P14, and P18 but could not yet be chemically identified. Another noteworthy band is observed at R_f_ 0.54 in the sample P32, displaying a %RSA of 93.2. This unidentified band exhibits potent antioxidant properties and serves as the primary contributor to the antioxidant activity of this sample.

Despite some commonalities across samples, the respective propolis extract’s antioxidant activity is related to its unique bioactive constituent profile. The identified compounds in WA propolis reflect some of the broad variety of chemical compounds commonly found in propolis; it is interesting to note, for example, that they have also been reported for propolis samples from other countries, such as Poland, Mexico, Brazil, China, and Turkey [29]. Similarly, although P10, P14, and P18 were collected from different regions in Western Australia, all three contained naringenin which was, in all three cases, one of the main constituents conferring medium to high antioxidant activity. Naringenin is indeed known to have many biological effects, such as antioxidant activity, but has also been reported to have anticancer and anti-inflammatory properties [40].

One of the most prominent bands in the HPTLC-DPPH assay of P29 was identified as luteolin. Taxifolin was also found in P29, but its antioxidant activity was not as high as that of luteolin. Luteolin is a tetrahydroxyflavone for which a wide range of pharmacological properties have been reported, among these, anti-inflammatory, antioxidant, neuroprotective, and analgesic effects [41]. Taxifolin, on the other hand, has also been found to have antioxidant, anti-inflammatory, and anti-microbial activities [42], although, in this study, its antioxidant activity towards DPPH was not as strong as that observed for luteolin.

4-HPAA, found in P28, also contributes to the antioxidant activity of its ethanolic extract. It is one of the major metabolites of polyphenols and is known to exert antioxidant and anti-inflammatory activities [43].

The presence of the identified compounds in Western Australian propolis warrants further research as studies on Brazilian propolis have shown the anti-tumour activity of compounds such as naringenin and luteolin. Other studies from Mexico have found naringenin and luteolin to have hypoglycaemic activities and to alleviate symptoms of diabetes mellitus in mice [44]. These studies underline the importance of further exploring those and other unidentified components of different Western Australian propolis samples and their potential for medical use.

## 4. Materials and Methods

### 4.1. Chemicals, Reagents, and Materials 

The chemicals and reagents used in this study were sourced as follows:

Folin and Ciocalteu’s phenol reagent 2N (F9252-1L), 2,4,6-tris(2-pyridyl)-1,3,5-triazine (TPTZ, 3682-35-7), iron (III) chloride hexahydrate (10025-77-1), and iron (II) sulphate heptahydrate (7782-63-0) were from Sigma Aldrich, Truganina, Australia; vanillin (121-33-5) was from Sigma-Aldrich, St. Louis, MO, USA; methanol (CH_3_OH, B.n. 19758725, 67-56-1) was from Scharlau, Barcelona, Catalonia, Spain; ethanol (64-17-5), anhydrous sodium carbonate (Na_2_CO_3_, 497-19-8) and aminoethyl diphenylborinate (524-95-8) were from Chem Supply, Port Adelaide, SA, Australia; toluene (108-88-3) was from APS Chemicals, Sydney, NSW, Australia; hydrochloric acid (7647-01-0) was from Asia Pacific Specialty Chemicals Limited, Seven Hills, NSW, Australia; gallic acid (149-91-7) was from Ajax Chemicals Limited, Sydney, NSW, Australia; 4′,5,7-trihydroxyflavanone (naringenin) (67604-48-2) was from Alfa Aesar, Lancashire, UK; 1,1-diphenyl-2-picrylhydrazyl (DPPH, 1898-66-4) was from Fluka AG, Buchs, St. Gallen, Switzerland; ethyl acetate (141-78-6) and formic acid (64-18-6) were from Ajax Finechem, Wollongong, NSW, Australia; sulfuric acid 98% (7664-93-9) was from Ajax Finechem, Wollongong, NSW, Australia, polyethylene glycol 400 (25322-68-3) was from PharmAust, Welshpool, WA, Australia; and HPTLC Silica gel 60 F254 Plates 10 × 20 cm was from Merck KGaA, Darmstadt, Hessen, Germany. Standards for the identification of the unknown bands were purchased from Combi-Blocks Inc. (San Diego, CA, USA): luteolin (491-70-3) and naringenin (480-41-1); from Sigma Aldrich (Castle Hill, Australia): p-hydroxyphenylacetic acid (156-38-7); from AK Scientific, Inc. (Union City, CA, USA): taxifolin (480-18-2) [22].

### 4.2. Samples 

**Figure 3:**
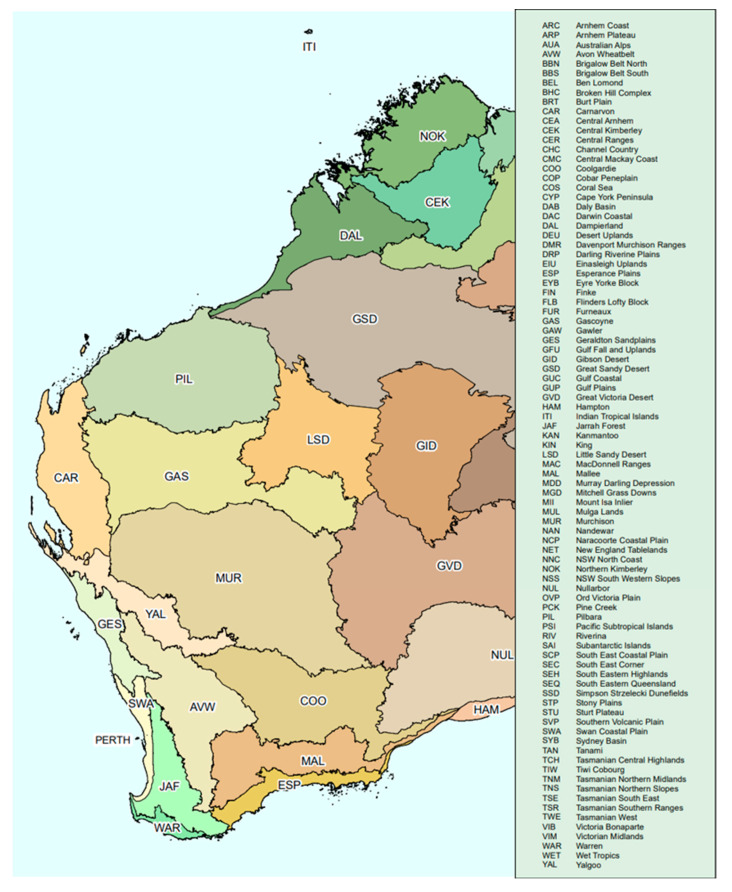
Collection sites of propolis. Interim Biogeographic Regionalisation for Australia, Version 7 [45,46].

In total, 32 Propolis samples were supplied as crude materials by local beekeepers from different regions of Western Australia (Table 6 and Figure 3). The samples were stored at 4 °C in falcon tubes before their sensorial characteristics and some basic physicochemical parameters were recorded, their antioxidant activity and total phenolic content were determined, and their HPTLC fingerprints were obtained.

For this, the 32 propolis samples were preliminarily extracted by placing 100 mg of propolis in a falcon tube followed by the addition of 21 mL of 70% ethanol. The suspensions were kept for 30 min at 40 °C in a heating oven (Memmert GmbH, Schwabach, Germany) and then placed in an ultrasonic bath (Unisonics Australia, Sydney, Australia) for 5 min. The extracts were collected in another falcon tube and stored at 4 °C until their total phenolic content (TPC) was determined (Section 4.7), and then the Ferric Reducing Antioxidant Power (FRAP) assay (Section 4.8) was carried out, and the preliminary HPTLC fingerprints (Section 4.9) were obtained (Table S1 and Figure S1). On this basis, six propolis samples (Table 1) were selected for a more in-depth analysis, whereas samples with either low TPC and FRAP results or close similarities in their HPTLC fingerprints were not analysed further.

### 4.3. Sensorial Analysis 

Each sample was subjected to a sensory analysis, including colour, aroma, and consistency at room temperature according to the recommendations of the Brazilian Ministry of Agriculture, which state that these characteristics depend on propolis’ respective botanical origin [25,47]. In brief, the Ministry of Agriculture distinguishes between balsamic and resinous aromas. Furthermore, propolis colours are classified as yellow, brown, green, and others and propolis consistency is categorised as either malleable or solid.

### 4.4. pH

The pH of the selected propolis samples (0.1 g in 7.5 mL of 70% EtOH) was measured at 25 °C with a calibrated Thermo Scientific Orion 3 Star pH Meter (Beverly, MA, USA) [47].

### 4.5. Oxidation Index 

A total of 0.2 g of raw propolis was dissolved in 5 mL of 70% ethanol and incubated for 1 h at room temperature. After the addition of 100 mL of deionised water, 1 mL of the resulting solution was aliquoted in a beaker and diluted with 40 mL of deionised water and 1 mL of 20% sulphuric acid (*v*/*v*). After stirring for 5 min, 5 µL of 0.1 M potassium permanganate was added, and, using a stopwatch, the time (in seconds) was recorded for the purple colour to fully disappear [25,48].

### 4.6. Propolis Extraction of Samples Selected for In-Depth Analysis

Raw propolis samples were removed from the refrigerator and immediately cut into small pieces. In total, 500 mg of propolis was placed in a falcon tube, and 3.5 mL of 70% ethanol was added. The suspension was heated for 30 min at 65 °C in a water bath (Industrial Equipment & Control PTY. LTD, Thornbury, VIC, Australia) [11] before being cooled down to room temperature. The cooled suspension was then placed in an ultrasonic bath (Unisonics Australia, Sydney, NSW, Australia) for 5 min [11]. The extract was collected in another falcon tube. The extraction protocol was repeated two more times and the extracts combined to yield a total extract volume of 10.5 mL. The suspension was centrifuged at RCF = 2086× *g* for 10 min at 4 °C [11] using a Sigma 2-16PK refrigerated benchtop centrifuge from John Morris Scientific, Chatswood, NSW, Australia. The supernatant was collected in a falcon tube and stored at 4 °C until further analysis. The residue from the centrifugation was transferred onto filter paper and dried at room temperature before the final constant weight was recorded to calculate the propolis concentration in each extract.

### 4.7. Determination of Total Phenolic Content (TPC) 

The total phenolic content (TPC) of the obtained ethanolic propolis extracts was determined using the Folin–Ciocalteu assay, and the findings were expressed as the mg gallic acid equivalent (GAE) per g of raw propolis. A standard curve was prepared using aqueous gallic acid solutions ranging from 0.03 mg/mL to 0.09 mg/mL. Around 1 mL of Folin–Ciocalteu reagent was diluted in 30 mL of deionised water before use. Then, 200 µL of each standard and 200 µL of each ethanolic propolis extract were mixed with 1 mL of Folin–Ciocalteu reagent. After 5 min, an 800 µL aqueous Na_2_CO_3_ solution (0.75% *w*/*v*) was added to all solutions [49]. The solutions were kept in the dark for 2 h at room temperature before their absorbance was measured at 760 nm using a Cary 60 UV-Vis Spectrophotometer from Agilent, Santa Clara, CA, USA. All samples were measured in triplicates and their TPC was calculated as follows:

(1) $$TPC\;Value\;of\;Sample\;\left(mg\;Gallic\;acid\right)=\frac{\left(\Delta Abs-intercept\right)}{slope}$$

### 4.8. Determination of Antioxidant Activity Using Ferric Reducing Antioxidant Power (FRAP) Assay 

The Ferric Reducing Antioxidant Power (FRAP) assay is a spectrophotometric analysis which is based on the reduction of a ferric tripyridyltriazine (Fe^III^-TPTZ) complex at a low pH [50]. The complex displays an intense blue colour at 620 nm [50] that is formed in the presence of antioxidants. The determination of FRAP activity of the ethanolic propolis extracts was carried out according to the protocol described by Almeida et al. [51] with minor modifications. Dilutions of 2 mM Ferrous sulphate (FeSO_4_ • 7 H_2_O) ranging from 200 µM to 1200 µM were used for the standard curve. Additionally, the standard concentrations of 600 µM and 840 µM were used as positive controls. The FRAP reagent was freshly prepared by mixing 10 mM TPTZ (dissolved in 40 mM HCl), 20 mM aqueous FeCl_3_ • 6 H_2_O, and 300 mM sodium acetate buffer (pH 3.6) in a ratio of 1:1:10 (*v*/*v*/*v*). The blank solution contained 300 mM aqueous sodium acetate buffer (pH 3.6), 40 mM HCl, and 20 mM aqueous FeCl_3_ • 6 H_2_O in a ratio of 10:1:1 (*v*/*v*/*v*). The reagent and blank solutions were incubated at 40 °C for 10 min prior to use.

In total, 20 µL of each standard or sample solution was pipetted into a 96-well plate (Greiner Bio-One 96-well Microplate Flat Bottom) and mixed with 180 µL of FRAP reagent or blank solution. The absorbance of the 96-well plate was scanned with a POLARstar Optima Microplate Reader (BMG Labtech, Ortenberg, BW, Germany) at 620 nm empty, after adding all reagents (t = 0) and also after storing the plate for 30 min in the dark at room temperature. The FRAP antioxidant activity was determined by the interpolation of the standard curve and expressed as mmol Fe^2+^ equivalent (FE)/kg raw propolis using the following equation:

(2) $$FRAP\;Value\;of\;Sample\;\left(µM\;Fe\left(II\right)\right)=(\frac{\left(\Delta Abs-intercept\right)}{slope})$$

### 4.9. HPTLC Fingerprinting 

#### 4.9.1. Standard Solution and Reagent Preparations 

A standard stock solution of gallic acid (2 mg/mL) in methanol and a reference solution of 0.5 mg/mL of 4′,5,7-trihydroxyflavanone in methanol were prepared. The derivatisation reagent for DPPH-HPTLC analysis was prepared by dissolving 40 mg DPPH in 10 mL of a methanol/ethanol solution (1:1), and after filtration, the solution was stored in an amber glass bottle until use. The vanillin spraying reagent was prepared by diluting 1 g of vanillin in 100 mL of ethanol (96%) to which 2 mL of sulphuric acid 98% was added dropwise. A natural product derivatisation reagent was prepared by dissolving 1 g of 2-aminoethyl diphenylborinate in 100 mL of methanol. The PEG solution was prepared by dissolving 5 g of polyethylene glycol 400 (PEG) in 100 mL of ethanol (96%).

#### 4.9.2. Sample Application 

To obtain the HPTLC fingerprints of the propolis samples, 4 µL of 4′,5,7-trihydroxyflavanone in methanol (0.5 mg/mL) as a standard and 3 µL of the ethanolic extracts of P10, P14, P18, and P29, as well as 4 µL of the ethanolic extracts of P28 and P32, were applied at a rate of 150 nL/s, 8 mm from the bottom of the HPTLC plate, using a semi-automated HPTLC application device (Linomat 5, CAMAG, Muttenz, BL, Switzerland).

#### 4.9.3. Development 

The development of each silica gel 60 F_254_ HPTLC plate (glass plates 20 cm × 10 cm and 10 × 10 cm, Merck, Darmstadt, Germany) was performed in a saturated (33% relative humidity), automated development chamber (ADC 2, CAMAG, Muttenz, BL, Switzerland) using toluene/ethyl acetate/formic acid (6:5:1, *v*/*v*/*v*) as the mobile phase [16]. The plates were pre-saturated with the mobile phase for 5 min, developed to a distance of 70 mm at room temperature, and dried for 5 min. Each plate was documented using an HPTLC imaging device (TLC Visualizer 2, CAMAG, Muttenz, BL, Switzerland) under white light and at 254 nm and 366 nm. The chromatographic images were analysed by a specialised HPTLC software (VisionCATS v3.1, CAMAG) [52].

#### 4.9.4. Derivatisation 

HPTLC plates were either derivatised with

1. A 3 mL vanillin sulfuric acid spraying reagent (VSA) using the yellow nozzle on level 3 (TLC Derivatiser, CAMAG, Muttenz, BL, Switzerland) followed by heating on a TLC Plate Heater III (CAMAG) at 115 °C for 5 min;
2. A 3 mL natural product derivatisation reagent (NP) using the green nozzle on level 3 (TLC Derivatiser, CAMAG, Muttenz, BL, Switzerland) followed by heating at 40 °C for 5 min, followed with derivatisation with 2 mL of PEG solution using the blue nozzle on level 2 (TLC Derivatiser, CAMAG, Muttenz, BL, Switzerland) and heating at 40 °C for 5 min.

### 4.10. Phenolic Constituent Identification in Propolis 

An HPTLC-derived database of 107 standards of mainly phenolic compounds was used to identify constituents in the investigated ethanolic propolis extracts following a protocol established by Lawag et al. [22]. In brief, the ethanolic propolis extracts were fingerprinted by HPTLC analysis using the various conditions outlined in Section 4.9. The results (i.e., R_f_ values, colour hues, UV-Vis, and fluorescence λ_max_ and λ_min_ prior to and after derivatisation) were matched with standards in the database [22]. Potential matches were confirmed by another HPTLC analysis where identified standards were run alongside the respective ethanolic propolis samples.

### 4.11. HPTLC-DPPH Analysis 

1,1-Diphenyl-2-picrylhydrazyl (DPPH) is a stable free radical which has a deep violet colour seen in a characteristic absorption band at 520 nm. In the presence of antioxidants, DPPH is reduced, resulting in a colour change to pale yellow, which forms the basis for the determination of antioxidant activity [53]. The same chromatographic device and parameters as described in Section 4.9 were used to perform the HPTLC-DPPH analysis with the ethanolic propolis extracts. In this case, however, samples P10, P14, P18, P29, and P32 were diluted at 1:5 with 70% ethanol, and sample P28 was diluted at 1:7 with 70% ethanol. P32 had an application volume of 3 µL, and for all other samples, 2 µL was applied. After development, the plates were derivatised with 3 mL of 0.4% DPPH solution (green nozzle, level 3). The images of the plates were taken with the TLC Visualizer 2, CAMAG in transmittance mode at white light after 120 min. Following a method developed by Lawag et al. [22] to semi-quantitatively determine antioxidant activity, each antioxidant band in the various propolis extracts was scanned at 517 nm to obtain its peak profiles. Colours for each band were derived from their respective RGB values which were then converted into corresponding hue values (H°S) [22]. H°P represents the hue value of unreacted DPPH on the plate (n = 10). Gallic acid was used as a positive control producing a maximum hue value of H°G = 40° [16]. The antioxidant activity of each band was then expressed as a percentage of DPPH radical scavenging activity (%DPPH RSA) using the following equation for bands with H°S < 40°:

(3) $$\%DPPH\;RSA=\left(\frac{\left(H{}^{\circ}S+H{}^{\circ}P\;\right)}{\left(H{}^{\circ}P+H{}^{\circ}G\right)}\right)*100$$

The antioxidant activity of each band was then categorised based on its intensity in terms of no activity (0.0% DPPH RSA), low activity (1.0–33.3% DPPH RSA), medium activity (33.4–66.6% DPPH RSA), and high activity (66.7–100.0% DPPH RSA) [22].

### 4.12. Data Analysis and Statistics 

Where appropriate, experiments were performed in triplicate, and the results were expressed as mean ± standard deviation. An analysis of variance (ANOVA) was performed to determine whether there was a significant difference (*p* < 0.05) in TPC and FRAP activity of the different ethanolic propolis extracts.

## 5. Conclusions

While research into the antioxidant activity of propolis and the identification of bioactive constituents is growing around the world, to our knowledge, there are currently no studies on Western Australian propolis specifically and only a few that have, to date, investigated Australian propolis. Thus, the findings of strong antioxidant properties in Western Australian propolis and the identification of some antioxidant constituents, such as luteolin and naringenin, address an important gap in current knowledge. As the biological activity of propolis depends on its chemical composition and is therefore mainly dependent on the plant species involved in its production, Western Australian propolis differs in its appearance, composition, and antioxidant activity from propolis samples collected elsewhere. Due to Western Australia’s rich and often endemic flora, more research should be carried out in the future to identify additional bioactive constituents, ideally using high-end hyphenated identification techniques such as HPLC-MS/MS or NMR analysis, and to determine their in vitro and in vivo effects to underpin a future potential use of Western Australian propolis as a natural product in complementary medicine.

## Figures and Tables

**Table 1:** Sensorial attributes of selected propolis samples.

Sample Name	Sample Picture	Colour	Aroma	Consistency
P29	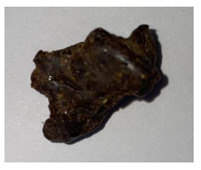	Brown	Resinous	Malleable
P10	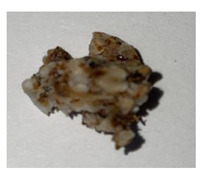	Brown, White	Resinous	Malleable
P14	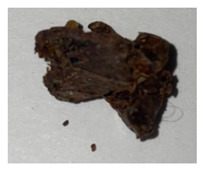	Brown	Resinous	Malleable
P18	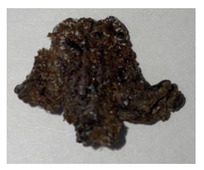	Brown	Resinous	Malleable
P28	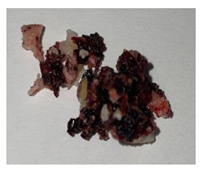	Red	Resinous	Malleable
P32	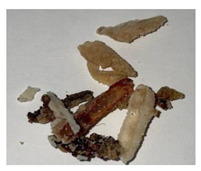	Red, Brown	Resinous	Malleable

**Table 2:** Average pH value of ethanolic propolis extracts, oxidation index, and extraction yield.

Propolis Sample	P29	P10	P14	P18	P32	P28
Average pH	4.60 ± 0.26 ^a,b^	4.40 ± 0.17 ^a^	4.20 ± 0.06 ^a^	4.10 ± 0.06 ^a^	4.90 ± 0.00 ^b^	4.40 ± 0.15 ^a^
Oxidation Index [s]	4.20 ± 0.72 ^a^	5.00 ± 1.00 ^a^	10.4 ± 0.51 ^b^	11.8 ± 1.08 ^b^	30.9 ± 2.17 ^c^	21.9 ± 2.05 ^d^
Extraction Yield	41.0%	21.5%	22.8%	32.1%	0.10%	24.4%

Different superscript letters in the same row denote significant differences (ANOVA, *p* < 0.05).

**Table 3:** Mean total phenolic content (TPC) and FRAP antioxidant activity of different Western Australian ethanolic propolis extracts.

Propolis Samples	P29	P10	P14	P18	P32	P28
TPC (mg GAE/g raw propolis)	59.3 ± 0.93 ^a^	27.3 ± 0.2 ^b^	31.0 ± 0.55 ^c^	50.1 ± 1.76 ^d^	9.26 ± 0.20 ^e^	39.3 ± 0.46 ^f^
FRAP (mmol Fe^2+^/kg raw propolis)	53.8 ± 4.43 ^a^	9.72 ± 0.59 ^b^	24.8 ± 3.84 ^c^	33.7 ± 4.96 ^c^	8.68 ± 0.11 ^d^	10.3 ± 0.99 ^b^

Different superscript letters in the same row denote significant differences (ANOVA, *p* < 0.05).

**Table 4:** Identification of unknown bands.

Sample	R_f_ Value Sample	Hue Value [°] Sample Der254	Colour Band Der254	Hue Value [°] Sample Der366	Colour Band Der366	Match	R_f_ Value Match	Hue Value [°] Match Der254	Colour Band Der254	Hue Value [°] Match Der366	Colour Band Der366
P10	0.565	139		138		Naringenin	0.584	138		166	
P14	0.577	138		182		Naringenin	0.584	138		166	
P18	0.561	140		152		Naringenin	0.584	138		166	
P28	0.475	138		217		4-HPAA	0.454	139		196	
P29	0.355	138		187		Taxifolin	0.373	119		49.6	
P29	0.415	138		183		Luteolin	0.411	130		183	
P29	0.470	135		180		Luteolin	0.489	138		200	

**Table 5:** Percentage DPPH RSA antioxidant activity of individual bands.

Sample	R_f_	Hue Value [°]	Colour Band	%RSA	Category	Matches of Section 2.6
Baseline	NA	340		0.00	0	NA
Gallic acid	0.285	37.9		96.5	+++	NA
P10	0.479	343		5.00	+	NA
	0.554	31.8		86.3	+++	Naringenin
	0.630	22.4		70.7	+++	NA
P14	0.552	2.70		37.8	++	Naringenin
	0.638	24.4		74.0	+++	NA
P18	0.565	27.2		78.7	+++	Naringenin
	0.633	36.9		94.8	+++	NA
P28	0.454	352		20.0	+	4-HPAA
	0.537	39.5		99.2	+++	NA
	0.605	35.9		93.2	+++	NA
	0.663	10.4		50.7	++	NA
P29	0.370	345		8.33	+	Taxifolin
	0.400	2.40		37.3	++	Luteolin
	0.454	13.0		55.0	++	Luteolin
	0.532	39.8		99.7	+++	NA
	0.615	357		28.3	+	NA
P32	0.542	35.9		93.2	+++	NA
	0.610	8.40		47.3	++	NA
	0.673	346		10.0	+	NA

**Table 6:** Propolis samples and selected samples for in-depth analysis.

Propolis Sample	Location	Biogeographical Region * (Figure 3)	Flora	In-Depth Analysis (Marked with X)
P01	Bullsbrook	SWA	Native bushland	
P02	Gidgegannup	JAF	Capeweed (*Arctotheca calendula*)	
P03	Fremantle	SWA	Bushland	
P04	Kalamunda	SWA	Mixed bushland	
P05	Viveash	SWA	Golden Wattle (*Acacia pycnantha*), Marri (*Corymbia calophylla*), River Red Gum (*Eucalyptus camaldulensis*), various other Eucalypts	
P06	Viveash	SWA	Golden Wattle (*Acacia pycnantha*), Marri (*Corymbia calophylla*), River Red Gum (*Eucalyptus camaldulensis*)	
P07	Viveash	SWA	Urban flora	
P08	Viveash	SWA	Urban flora, River Red Gum (*Eucalyptus camaldulensis*)	
P09	Viveash	SWA	Urban flora, Golden Wattle (*Acacia pycnantha*), various Eucalypts	
P10	Wellstead	ESP	Native bush, Canola (*Brassica nabus*)	X
P11	Wellstead	ESP	Native bush, Canola (*Brassica nabus*)	
P12	Albany + Muchae	JAF	Wildflowers	
P13	Swan View	SWA	Mixed urban flora	
P14	Gidgegannup	JAF	Dryandra (*Banksia sessilis*)	X
P15	Swan View	SWA	N/A	
P16	Brentwood	SWA	Urban flora	
P17	Wooroloo	JAF	N/A	
P18	Two Rocks	SWA	N/A	X
P19	Bullsbrook	SWA	N/A	
P20	Roleystone	JAF	Citrus and other orchard fruit trees	
P21	Medina	SWA	N/A	
P22	Bedfordale	SWA	Marri (*Corymbia calophylla*), Jarrah (*Eucalyptus marginata*)	
P23	Baldivis	SWA	Parrot Bush (*Banksia sessilis*), various Eucalypts	
P24	Armadale	SWA	Various Eucalypts, Capweed (*Arctotheca calendula*)	
P25	Morangup	JAF	Native bushland, various Eucalypts, pine trees (*Pinus* spp.)	
P26	Bindoon, Chittering	JAF	Mixed flora	
P27	Bindoon, Chittering	JAF	Mixed flora	
P28	Ninghan station	AVW	N/A	X
P29	Yanchep	SWA	Thick-Leaved Fan-Flower (*Scaevola crassifolia*)	X
P30	Yanchep	SWA	Thick-Leaved Fan-Flower (*Scaevola crassifolia*)	
P31	Yanchep	SWA	N/A	
P32	Ninghan station	AVW	N/A	X

* SWA = Swan Coastal Plain; JAF = Jarrah Forest; ESP = Esperance Plains.

## Data Availability

All data are presented in the article and Supplementary Materials.

## Supplementary Materials

The following supporting information can be downloaded at: https://www.mdpi.com/article/10.3390/plants13141919/s1, Figure S1: HPTLC fingerprints: transmittance at 254 nm (a) and 366 nm (b) after development. Transmittance at white light (c) and 366 nm (d) after derivatisation with VSA; (b) 366 nm after derivatisation with VSA; Table S1: total phenolic content and antioxidant activity of 32 preliminary Western Australian ethanolic propolis extracts; Table S2: spectra overlays with UV Vis and fluorescence after derivatisation.
