# Supplementary material for: Physicochemical Properties, Antioxidant Activity, and High-Performance Thin-Layer Chromatography Profiling of Propolis Samples from Western Australia
Source: Plants (Basel). 2024 Jul 11;13(14):1919. doi: 10.3390/plants13141919 (PMC11281206; doi:10.3390/plants13141919)
Supplement: Supplementary file 1 [file plants-13-01919-s001.zip › plants-3038201-supplementary.pdf]

Supplement:

Physicochemical Properties, Antioxidant Activity and Phytochemical Profiling of Propolis Samples from Western Australia

Table Supplement S1. Total phenolic content and antioxidant activity of 32 preliminary Western Australian ethanolic propolis extracts.

TPC (mg GAE/ 100 g raw propolis									
**Samples**	**P01**	**P02**	**P03**	**P04**	**P05**	**P06**	**P07**	**P08**	**P09**
Mean	327.1	209.67	299.38	1127.39	212.71	187.1	156.17	222.45	206.57
**Samples**	**P10**	**P11**	**P12**	**P13**	**P14**	**P15**	**P16**	**P17**	**P18**
Mean	1178.54	1125.32	194.77	286.09	1452.15	550.56	250.24	230.75	2008.96
**Samples**	**P19**	**P20**	**P21**	**P22**	**P23**	**P24**	**P25**	**P26**	**P27**
Mean	800.71	262.44	243.99	771.06	354.17	790.27	1377.58	502.03	694.75
**Samples**	**P28**	**P29**	**P30**	**P31**	**P32**				
Mean	4863.20	3355.30	3582.67	86.13	4455.62				
FRAP (mmol Fe^{2+}/kg raw propolis									
**Samples**	**P01**	**P02**	**P03**	**P04**	**P05**	**P06**	**P07**	**P08**	**P09**
Mean	23.23	17.00	9.36	97.84	15.99	16.10	12.13	1.21	16.08
**Samples**	**P10**	**P11**	**P12**	**P13**	**P14**	**P15**	**P16**	**P17**	**P18**
Mean	25.81	75.25	9.42	11.29	66.13	19.09	6.66	5.11	98.97
**Sample**	**P19**	**P20**	**P21**	**P22**	**P23**	**P24**	**P25**	**P26**	**P27**
Mean	23.97	7.70	4.96	33.89	10.26	29.49	92.13	31.22	39.93
**Sample**	**P28**	**P29**	**P30**	**P31**	**P32**				
Mean	69.71	1174.55	1141.99	32.90	64.49				

Figure Supplement S1. HPTLC Fingerprints: transmittance at 254 nm (a), 366 nm (b) after development. Transmittance at white light (c) and 366 nm (d) after derivatization with VSA.

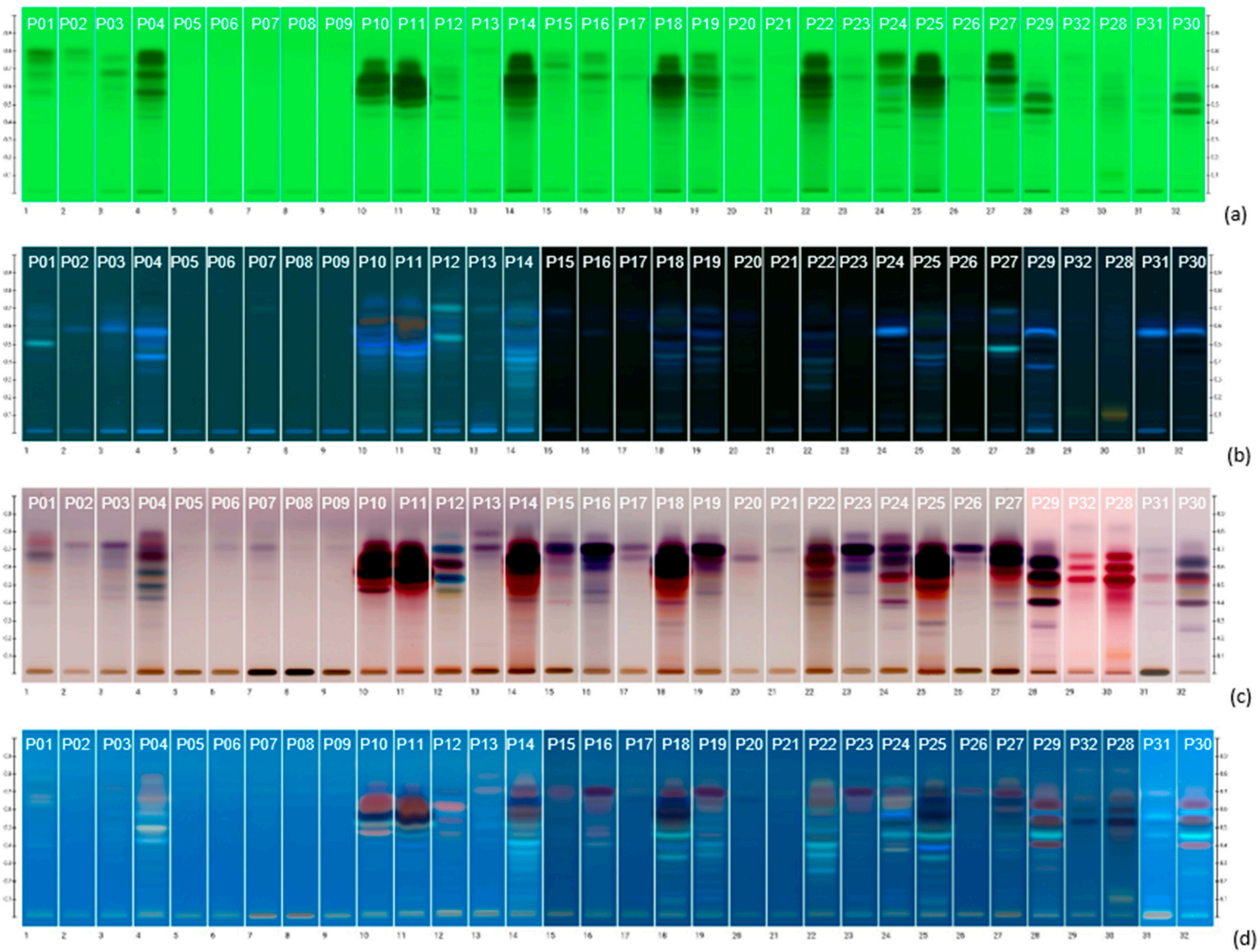
P01 P02 P03 P04 P05 P06 P07 P08 P09 P10 P11 P12 P13 P14 P15 P16 P17 P18 P19 P20 P21 P22 P23 P24 P25 P26 P27 P29 P32 P28 P31 P30
(a)
P01 P02 P03 P04 P05 P06 P07 P08 P09 P10 P11 P12 P13 P14 P15 P16 P17 P18 P19 P20 P21 P22 P23 P24 P25 P26 P27 P29 P32 P28 P31 P30
(b)
P01 P02 P03 P04 P05 P06 P07 P08 P09 P10 P11 P12 P13 P14 P15 P16 P17 P18 P19 P20 P21 P22 P23 P24 P25 P26 P27 P29 P32 P28 P31 P30
(c)
P01 P02 P03 P04 P05 P06 P07 P08 P09 P10 P11 P12 P13 P14 P15 P16 P17 P18 P19 P20 P21 P22 P23 P24 P25 P26 P27 P29 P32 P28 P31 P30
(d)

Table Supplement S2. Spectra overlays with UV Vis and Fluorescence after Derivatisation.

Sample with Standard	HPTLC fingerprints	Fluorescence overlay	UV Vis overlay
Sample P29 with Luteolin	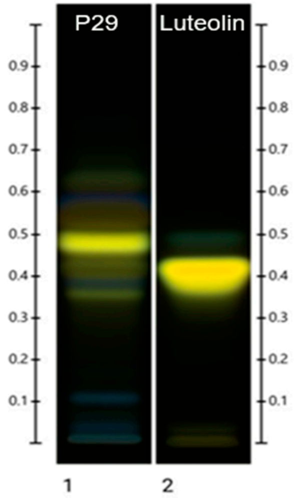	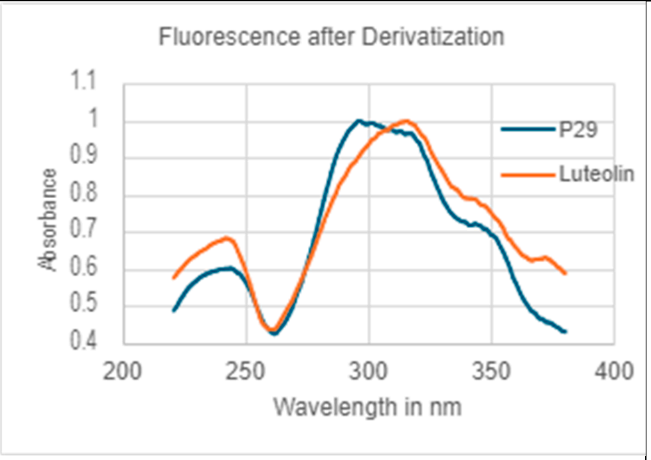	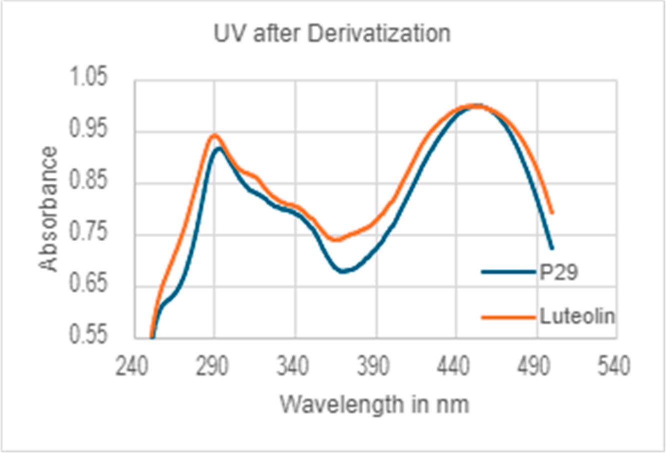
Sample P29 with Taxifolin	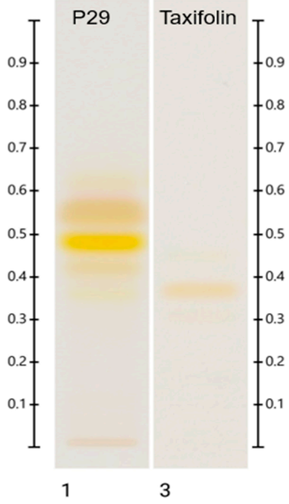	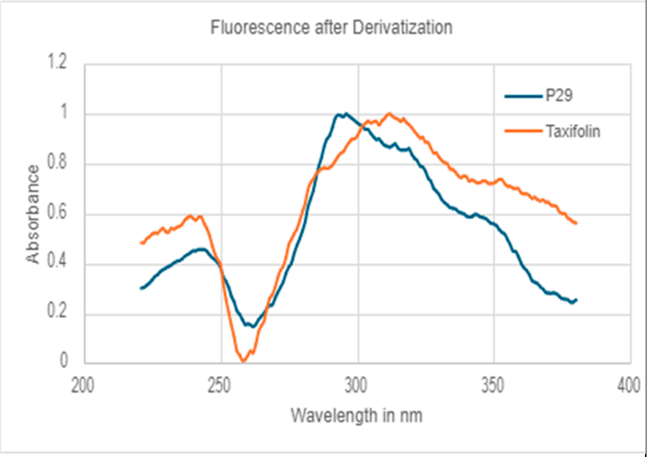	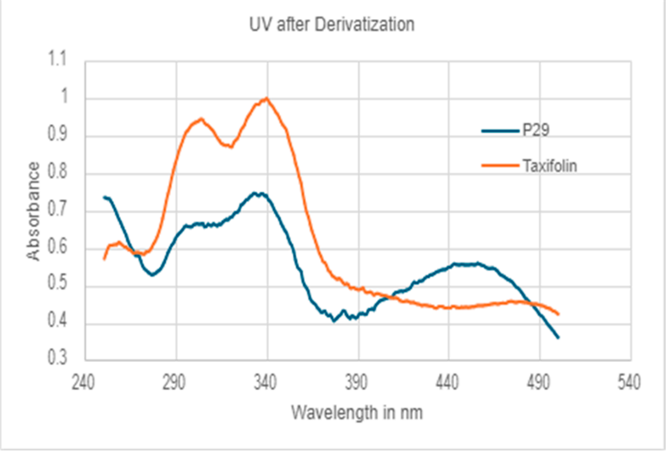

<table>
<tr>
<td>Sample P18 with Naringenin</td>
<td>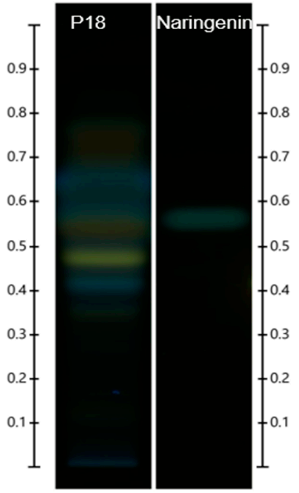
</td>
<td>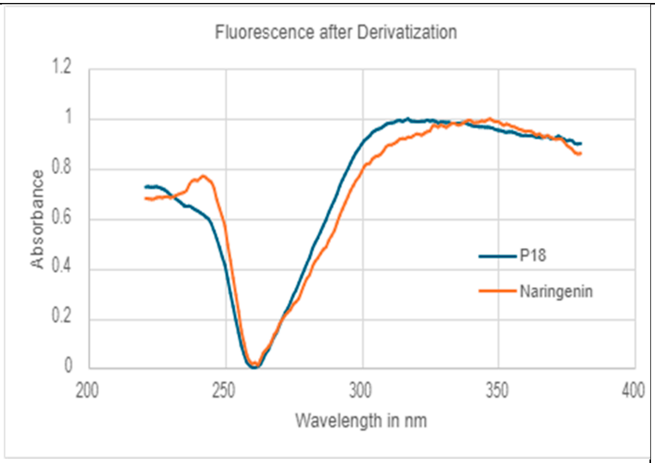
</td>
<td>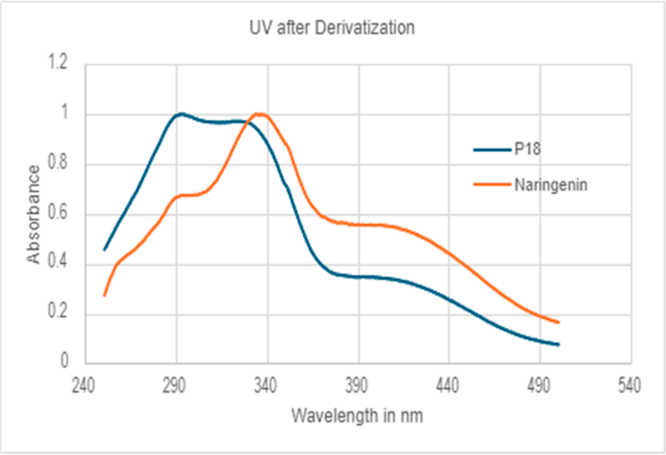
</td>
</tr>
<tr>
<td>Sample P14 with Naringenin</td>
<td>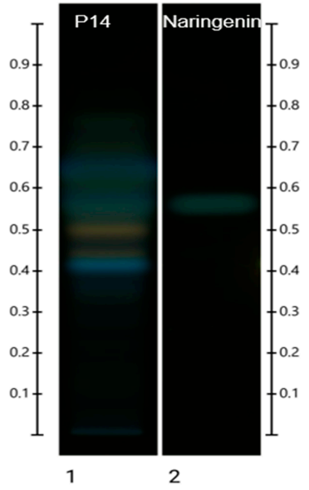
</td>
<td>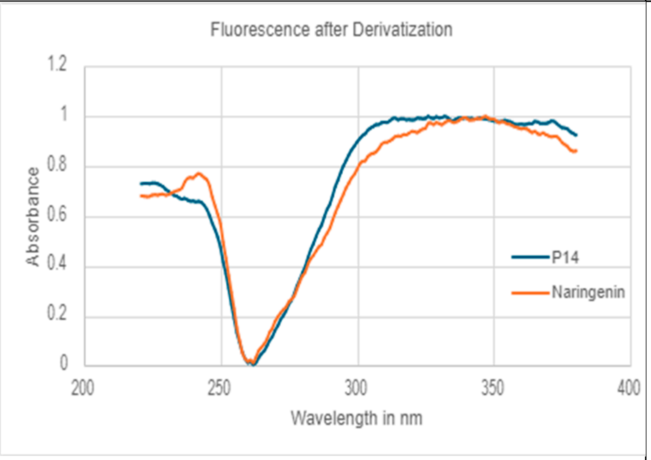
</td>
<td>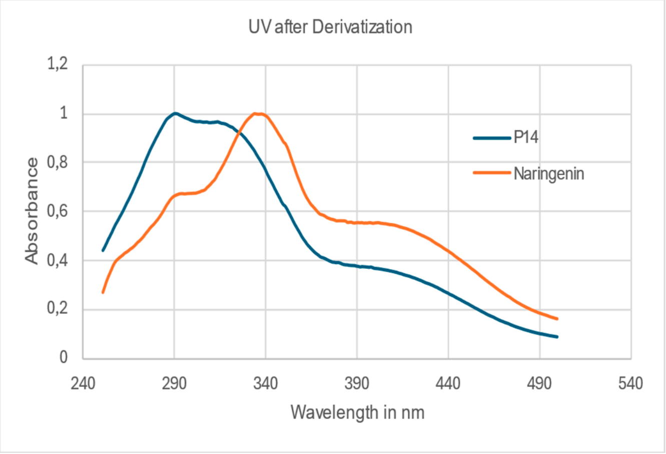
</td>
</tr>
</table>

<table>
<tr>
<td>Sample P10 with Naringenin</td>
<td>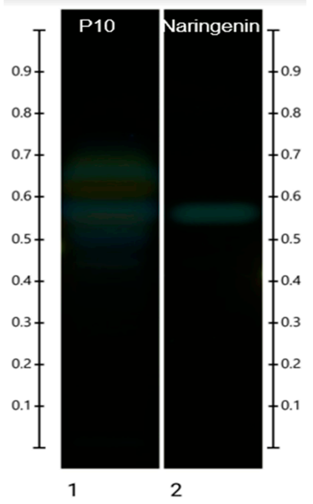
</td>
<td>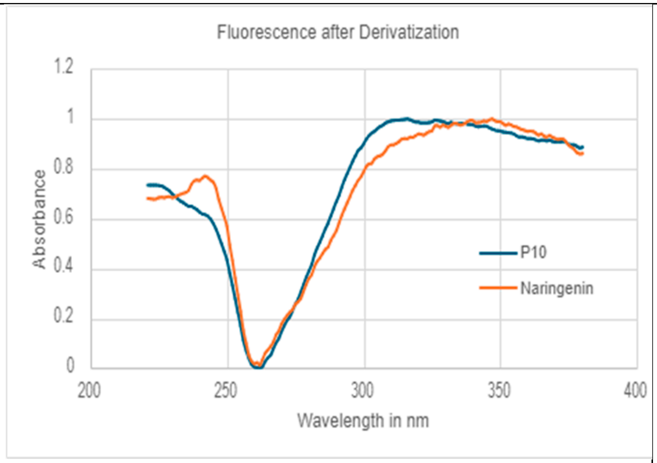
</td>
<td>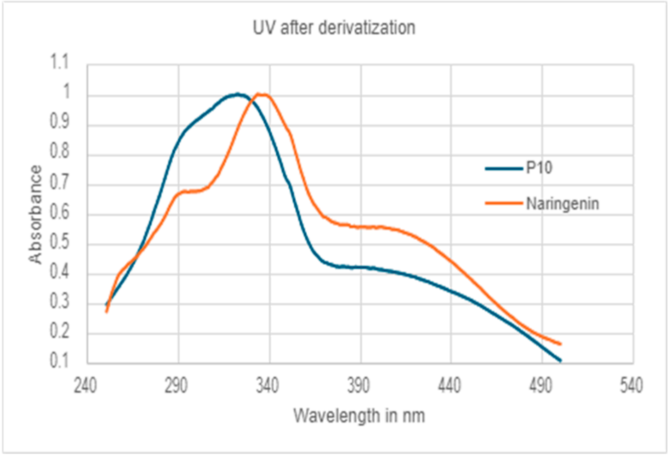
</td>
</tr>
<tr>
<td>Sample P28 with 4-HPAA</td>
<td>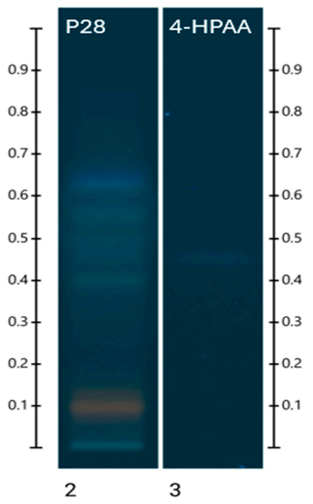
</td>
<td>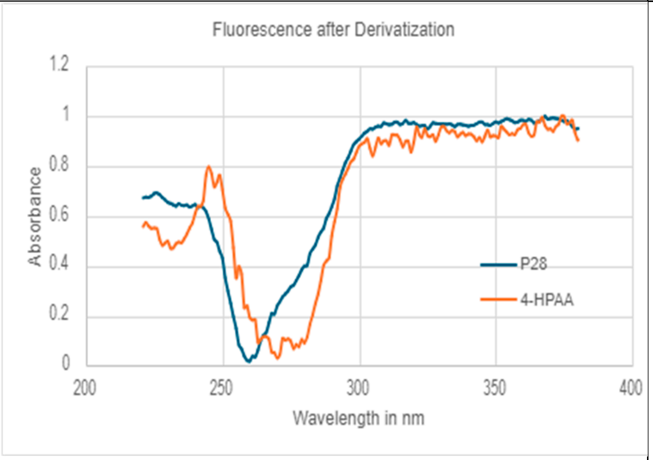
</td>
<td>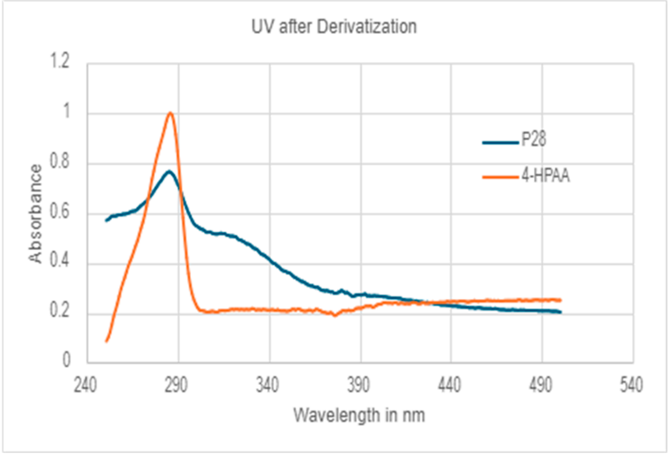
</td>
</tr>
</table>
